# The association between multi-disciplinary staffing levels and mortality in acute hospitals: a systematic review

**DOI:** 10.1186/s12960-023-00817-5

**Published:** 2023-04-20

**Authors:** Chiara Dall’Ora, Bruna Rubbo, Christina Saville, Lesley Turner, Jane Ball, Cheska Ball, Peter Griffiths

**Affiliations:** 1 School of Health Sciences, University of Southampton, Highfield Campus, Southampton, SO17 1BJ UK; 2 NIHR Applied Research Collaboration Wessex, University of Southampton, Southampton, UK; 3 Dorset County Hospital NHS Foundation Trust, Dorchester, UK

**Keywords:** Staffing, Hospital mortality, Workforce

## Abstract

**Objectives:**

Health systems worldwide are faced with the challenge of adequately staffing their hospital services. Much of the current research and subsequent policy has been focusing on nurse staffing and minimum ratios to ensure quality and safety of patient care. Nonetheless, nurses are not the only profession who interact with patients, and, therefore, not the only professional group who has the potential to influence the outcomes of patients while in hospital. We aimed to synthesise the evidence on the relationship between multi-disciplinary staffing levels in hospital including nursing, medical and allied health professionals and the risk of death.

**Methods:**

Systematic review. We searched Embase, Medline, CINAHL, and the Cochrane Library for quantitative or mixed methods studies with a quantitative component exploring the association between multi-disciplinary hospital staffing levels and mortality.

**Results:**

We included 12 studies. Hospitals with more physicians and registered nurses had lower mortality rates. Higher levels of nursing assistants were associated with higher patient mortality. Only two studies included other health professionals, providing scant evidence about their effect.

**Conclusions:**

Pathways for allied health professionals such as physiotherapists, occupational therapists, dietitians, pharmacists, to impact safety and other patient outcomes are plausible and should be explored in future studies.

**Supplementary Information:**

The online version contains supplementary material available at 10.1186/s12960-023-00817-5.

## Introduction

Having enough healthcare workers with the right skills is essential for maintaining patient safety and quality of care. Nonetheless, several health systems face critical shortages of staff either due to short supply or economic constraints, or both [1-3]. Despite absolute staff numbers increasing in many countries [4], staff workload has also increased, in part due to increase in patient volumes, ageing populations with more complex health conditions, meaning that the healthcare staff shortages persists.

The evidence that adequate staffing levels are important for good patient outcomes is extensive, but it has focused primarily on nursing. Several reviews have concluded that when patients are exposed to higher levels of registered nursing staff, the risk of dying while in hospital or soon after discharge is lower [5-8]. Despite the predominance of observational evidence, careful analysis supports a conclusion that a causal relationship is both plausible and likely [5, 6]. This has led a number of countries to introduce policies that mandate safe staffing ratios for nursing hospital services [9-12], but such policies have not extended to other healthcare professional groups.

Nonetheless, the healthcare workforce is made up of many different professional groups. Of all the healthcare professional groups, patients are most exposed to nursing staff when in hospital [13], but nurses are not the only professionals who interact with patients and staffing levels of other staff groups are also likely to influence the quality and safety of care. The focus purely on nurse staffing is thus a problem as there is potential for bias in effect estimates. If studies do not account for other occupational groups, an observed association between nurse staffing and patient mortality could be partly or wholly due to an effect of other occupational groups [14].

Evidence that could drive policy around other staffing groups, including pharmacists, physiotherapists, occupational therapists, dietitians, speech therapists, and podiatrists is sparse [15, 16]. Although there is more research on patient outcomes and physician staffing [17], we are not aware of any comprehensive systematic review synthesising evidence around the impact of staffing levels across multi-disciplinary teams. Therefore, the aim of this systematic review was to synthesise the evidence on the relationship between nurse and other occupational groups staffing levels and the risk of patients dying after being admitted to hospital.

## Methodology

### Eligibility criteria

We included quantitative or mixed methods studies with a quantitative component exploring the association between multi-disciplinary hospital staffing levels and mortality. We considered only studies that explored multivariable associations for more than one staffing group simultaneously and which included or adjusted for nurse staffing levels, as the causal influence of nurse staffing is well supported and so omission of this as a variable from other studies is likely to be a critical source of bias. We excluded studies that reported on one staffing group only, including studies exclusively exploring the mix of workers or substitutions within a single occupational group, for example a study considering only registered nurses and nursing assistants, or physicians and physician assistants would not be included. Due to the absence of previous reviews on the topic and due to limited knowledge around the depth and breadth of this body of evidence, no publication date restrictions were applied.

Studies that reported on all-cause or disease-specific mortality (or survival) in hospital or within 30 days of admission were included. Studies conducted in hospitals providing acute care were eligible for inclusion. We excluded studies conducted in the community, long-term or mental health facilities and studies that were only reported as conference abstracts.

### Study selection and data extraction

We performed the search in November 2021, following the registered systematic review protocol (PROSPERO registration CRD42020219869). We used Embase subject headings (Emtree) and Medical Subject Headings (MeSH) terms with additional free text keywords to search Embase, Medline, CINAHL, and the Cochrane Library. We hand-searched for additional articles by checking reference lists of included articles. While our search overall included three main facets “staffing groups and levels”, “hospital setting”, and “mortality” combined with the Boolean operator “and”, the exact search terms varied according to each database specific search functions. The full search strategy is available as Additional file 1: File S1.

One reviewer de-duplicated and assessed titles and abstracts for eligibility. Full text was obtained for all relevant studies and for those where there was uncertainty on eligibility. These were assessed independently by two reviewers. Manuscripts with uncertain eligibility after full text review were discussed with all co-authors to reach a consensus.

We used a standardised data extraction form, developed a priori in Excel. Two reviewers independently extracted data on publication (authors, title, and year and country of publication), study characteristics (design, data collection period, data sources, number of hospitals/units/patients included), measures of staffing levels (staff groups and definitions), outcomes including how they were measured, methodology (level of aggregation, type of data analyses), and findings (estimates with precision measures).

### Risk of bias assessment

We adapted the risk of bias assessment tool developed for studies of the association between healthcare staffing and outcomes [18]. This was based on the framework for assessment of quantitative studies reporting correlations and associations in the National Institute for Health Care Excellence (NICE) guidance for reviews in Public Health guidance [19]. The tool assesses the study’s internal and external validity separately. For each criterion, a rating of strong was assigned when the method adopted was likely to minimise bias, a rating of moderate where items lacked clarity or the methods did not address all likely sources of potential bias, or rating of weak where significant sources of bias might arise. A blank checklist is attached as supplementary material (Additional file 1: File S2). Two reviewers independently assessed all manuscripts included in the review for risk of bias. There was a percentage agreement of 92% and the Cohen’s kappa was 0.58, indicating moderate agreement, with 100% agreement reached after the moderation process. Disagreements were discussed with all co-authors until a consensus was obtained.

### Synthesis

We performed a narrative synthesis of the evidence as we were unable to conduct a formal meta-analysis due to the lack of studies using similar measures of staffing that could be grouped, and due to the different combinations of staffing groups included in the individual studies. Where studies presented results for more than one statistical model, we reported relationships from the most complete model (i.e. adjusted for the largest number of occupational groups).

## Results

**Fig. 1:**
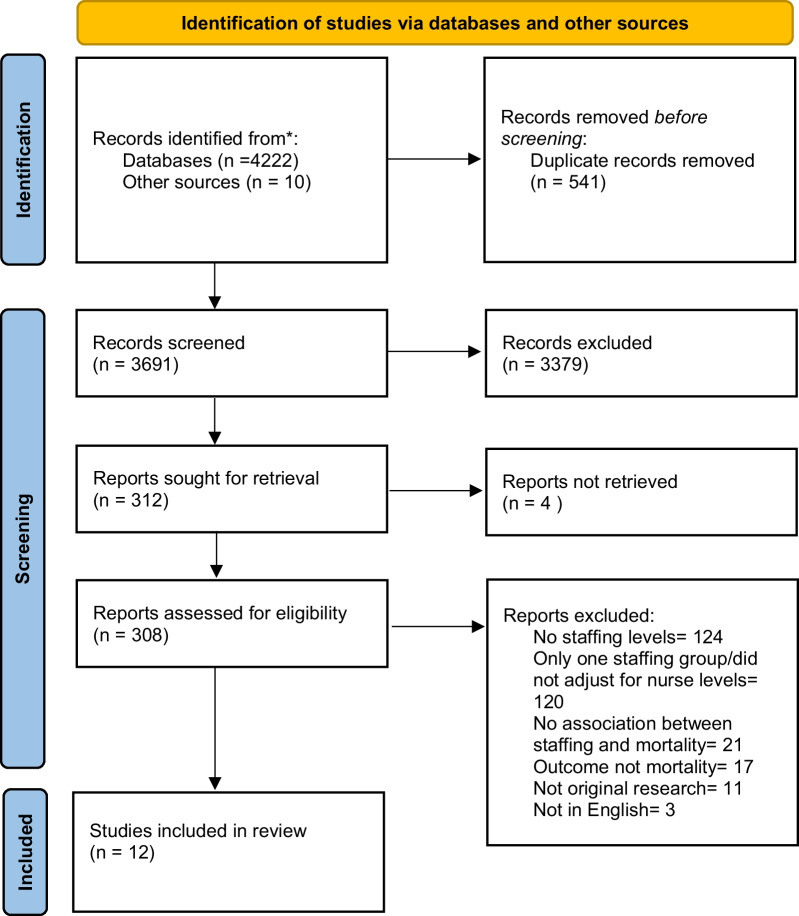
PRISMA flow diagram. From: Page MJ, McKenzie JE, Bossuyt PM, Boutron I, Hoffmann TC, Mulrow CD, et al. The PRISMA 2020 statement: an updated guideline for reporting systematic reviews. BMJ 2021;372:n71. https://doi.org/10.1136/bmj.n71. For more information, visit: http://www.prisma-statement.org/

We found 4222 abstracts, of which 3681 were screened after removal of 541 duplicates. We identified 312 potentially relevant studies were reviewed in full for eligibility, of which 12 met the inclusion criteria. Reasons for exclusion are listed in the PRISMA flowchart (Fig. 1).

### Study characteristics

All studies’ characteristics are reported in Table 1. Studies were published between 1999 and 2020 and included data from USA (5 studies), UK (2), South Korea (2), and one each from Denmark, France, and the Netherlands. Only one study was single-centred [20], with others including data from between four [21] and 3763 hospitals [22]. Ten studies were cross-sectional [20, 22-30] and two were cohort studies [21, 31].

**Table 1:** Summary of included studies' characteristics

Author(s), year	Country	Data collection period	Study design	Sample size	Data source
Bjerregard et al., 2020	Denmark	2015–2017	Retrospective cross-sectional	Hospitals = 1 Wards = 5 Patients = 135,728	• Hospital admissions = National Patient Register • Sociodemographic patient-level data = Statistics Denmark • Mortality = merging data from the NPR and cause of death register • Staffing levels = hospitals' payroll systems
Bond et al., 1999	USA	1992	Retrospective cross-sectional	Hospitals = 3763 Wards = N/A Patients = 23,879,998 (admissions)	Mortality = Medicare Hospital Mortality Information Average daily census, hospital variables and staffing levels = American Hospital Association (AHA) database
Checkley et al., 2014	USA	Not reported	Cross-sectional survey	Hospitals = 42 Wards = (ICUs) 69 Patients = N/A	Study questionnaire completed by ICU directors
Chung et al., 2018	South Korea	2009	Retrospective cross-sectional	Hospitals = 615 Wards = N/A Patients = 11,819	Patient admission = National Health Insurance claims data Patient and hospital data (including staffing levels) = Health Insurance Review and Assessment Service
Griffiths et al., 2016	UK	2009–2011	Retrospective cross-sectional	Hospitals = 137 (31 as subsample for ward-level analysis) Wards = 401 (only applies to ward-level analysis) Patients = 9,669,555 (medical admissions) and 9,302,292 (surgical admissions)	Workforce data = annual NHS staff census and RN4CAST survey for subsample of 31 Trusts Teaching status, bed occupancy and number of beds = annual estates and facilities statistics Patient data = Hospital Episode Statistics
Kim et al., 2020	South Korea	2014–2015	Retrospective cross-sectional	Hospitals = 43 Wards = N/A Patients = 67,927	National Health Insurance claims
Neuraz et al., 2015	France	2013	Longitudinal	Hospitals = 4 Wards = (ICUs) 8 Patients = 5,718	Patient admission = claims data used for billing inpatient stay nurse staffing levels = staff databases Staff qualifications and affiliations = HR database
Peelen et al., 2007	The Netherlands	2003–2005	Retrospective cohort study	Hospitals = N/A Wards = (ICUs) 28 Patients = 4,605	Patient admission = Dutch National Intensive Care Evaluation (NICE) registry Staffing levels and organisational factors = study questionnaire
Ricciardi et al., 2014	USA	2003–2008	Retrospective cross-sectional	Hospitals = N/A Wards = N/A Patients = 48,253,968 (patient discharges)	Patient discharge = Nationwide Inpatient Sample (NIS) of the Healthcare Cost and Utilization Project of the Agency for Healthcare Research and Quality Staffing levels and facility characteristics = American Hospital Association (AHA) Annual Survey database
Robertson et al., 1999	USA	1989–1991	Retrospective cross-sectional	Hospitals = 1791 (in 1989); 1784 (in 1990); 2133 (in 1991) Wards = N/A Patients = N/A	Patient mortality = Health Care Financing Administration (HCFA)'s Hospital Information Reports Staffing data and Hospital characteristics = American Hospital Association (AHA) Annual Survey Data Medicare Case Mix Index = Commission on Professional and Hospital Activities and HCIA Inc
Smith et al., 2007	USA	1999–2001	Retrospective cross-sectional	Hospitals = 214 Wards = N/A Patients = 1864	Mortality = Public Use Data File Socioeconomic and demographic data = Texas Hospital Discharge Public Use Data File Hospital-level data = Center for Medicare and Medicaid Services' Hospital Cost Report Information System and Provider of Services Staffing levels = American Hospital Association Survey
West et al., 2014	UK	1998	Retrospective cross-sectional	Hospitals = N/A Wards = (ICUs) 65 Patients = 38,168	Patient and outcome data = Intensive Care National Audit & Research Centre (ICNARC) Case Mix Programme Staffing levels = Audit Commission survey of ICUs Patient and hospital data = Health Insurance Review and Assessment Service

Author(s), year	Staff groups studied	Measure of staffing levels	Outcomes	Risk-adjustment	Level of aggregation for analysis
Bjerregard et al., 2020	• Nurses • Physicians • Nurse assistants	Staff HPPD	30-day mortality	Patient: Age Sex Highest education Income Ethnicity Disease severity (based on ICD-10) Comorbidities DRG price at discharge Mode of admission Department: Share of ambulatory patients to total patients Share of total activity related to emergency admission	Month-ward
Bond et al., 1999	• Administrators • Physicians • Medical residents • Registered nurses • Licensed practical vocational nurses • Physician assistants • Registered pharmacists • Medical technologists • Dietitians • Occupational therapists • Physical therapists • Respiratory therapists • Social workers • Departmental personnel	Staff per occupied bed	Mortality rates	Hospital: Occupancy rate Teaching affiliation Ownership (private nonprofit, private for-profit, public) Patient: Percentage of ICU days annual number of ER visits divided by average daily census Percentage of Medicaid patients	Year-Hospital
Checkley et al., 2014	• Intensivists • ICU fellows • ICU residents • Respiratory Therapists • Nurses • Physician Assistants • Nurse Practitioners	• Bed-to-nurse ratio • Trainee-to-bed ratio	Annual ICU mortality	Average APACHE II score ICU type Case volume Bed capacity 24-h intensivist coverage ICU organisation (open vs closed vs computerised patient order entry) Rounding practices (daily plan of care review vs multi-disciplinary rounding) Protocols	ICU
Chung et al., 2018	• Nurses • Physicians	Number of staff per 100 beds	In-hospital Mortality	Patient: Age Gender Insurance type Ischaemic stroke (yes vs no) Admission mode Receipt of surgery (yes vs no) Length of stay Medical costs Number of comorbidities	Hospital
Griffiths et al., 2016	• Registered Nurses • Doctors • Healthcare support workers (healthcare assistants and auxiliary nurses)	• Occupied bed per staff for hospital trust-level data • RN to patient ratio for ward-level data	In-patient mortality rates	Age Sex Elective status Socioeconomic deprivation Comorbidities Number of emergency admissions in the previous 12 months Reason for admission	Year-hospital; last shift worked-specialty of admission (subsample analysis)
Kim et al., 2020	• Nurses • Physicians	Number of beds per staff	In-hospital mortality post Percutaneous Coronary Intervention	Hospital: Ownership Location Number of PCI procedures Physician in charge of the ICU Patient: Sex Age Type of procedure Disease severity Insurance type Admission path Admission to the ICU	Hospital
Neuraz et al., 2015	• Nurses • Physicians	Patient-to-staff ratio	In-hospital mortality	ICU: Patient turnover Number of LSP Proportion of men Proportion of surgical cases Patient: SAPSII number of comorbidities	ICU-shift
Peelen et al., 2007	• Nurses • Intensivists	Staff per ICU bed	In-hospital mortality	Patient: Age Sex SAPS II score Number of dysfunctioning organ systems ICU: Number of admissions with severe sepsis Total number of admissions per year Number of ICU beds Number of hospital beds	ICU
Ricciardi et al., 2014	• Full-time registered nurses • Full-time physicians • Resident trainees (yes or no)	Staff per hospital bed	Inpatient mortality following non-elective admission	Patient: Age Sex Race Income level Payer Major diagnostic categories (subgroupings of diagnosis-related groups) Charlson comorbidity index score Hospital: Hospital bed size	Hospital
Robertson et al., 1999	• Administrators and assistant administrators • Physicians • Medical residents and interns • Registered nurses • Licensed practical and vocational nurses • Ancillary nursing personnel • Respiratory therapists • Respiratory therapy technicians • Radiographers and radiologic technologists • Radiation therapists • Nuclear medicine technologists • Other radiologic personnel • Medical technologists • Other laboratory personnel • Pharmacists • Occupational therapists • Physical therapists • Dieticians	Number of FTE staff employed per 100 adjusted admissions	30-day mortality	Hospital: Financial status Ownership Technology status Size Caregiver skill mix Patient: Aggregate severity of illness	Hospital-year
Smith et al., 2007	• Registered nurses • Licensed vocational nurses • Respiratory therapists	Staff-occupied bed ratio	In-hospital mortality	Hospital: High-volume hospital (performing at least 15 gastrectomies per year) Higher median of critical care beds Patient: Sex Age Education Metastasis Total gastrectomy Emergency admission Comorbid condition	Hospital
West et al., 2014	• Consultants • Intensivists (yes or no) • Nurses • Support staff (total number)	• FTE Registered nurses per bed • Consultant NHDs per bed	ICU mortality and in-hospital mortality	Patient: ICNARC model risk-adjustment (physiology model, including blood pressure, respiratory rate, oxygenation, and acid base disturbance, along with a range of other factors known to be associated with mortality, including age, past medical history, and source of admission to an ICU)	ICU

Patient sample sizes varied, ranging from 1864 [29] to 23,879,998 [22]. Studies with smaller samples focused on specific patient populations, e.g. patients who had a gastrectomy [29], or patients from ICU settings only [21, 31], whereas the larger studies included less specific populations of general medical and/or surgical patients.

All studies used bed-to-staff or staff-to-bed ratios to measure staffing levels, apart from two studies which reported staff-to-patient ratios [20, 21], and one study which reported the number of Full Time Equivalent staff employed per 100 adjusted admissions [28]. The majority of studies (*n* = 10) reported on all-cause mortality as the primary outcome, while two restricted on mortality after specific procedures (i.e. post-percutaneous coronary intervention [26] and post-gastrectomy [29]). All estimates from the multivariable models are reported in Table 2.

**Table 2:** Estimates from multivariable models

Author(s), year	Outcome	Level of aggregation	Estimate	RN staffing	Nurse assistant staffing	Physician staffing	Other staff groups
Bjerregard et al., 2020	All-cause mortality	Unit	Coefficient (robust SE)	Within HPPD 0.00271 (0.00169) Between HPPD − 0.008* (0.000669)	Within HPPD − 0.0118* (0.00557) Between HPPD − 0.00872* (0.00319)	Within HPPD − 0.01* (0.00372) Between HPPD 0.0113 (0.00576)	
Bond et al., 1999	All-cause mortality	Hospital	Slope/rate of change (95%CI)	Per occupied bed − 0.0026* (− 0.00456 to − 0.00064)	Per occupied bed 0.0047* (0.00176 to 0.00764)	Per occupied bed − 0.0017* (− 0.00562 to − 0.00222) Residents per occupied bed: − 0.0085* (− 0.01301 to − 0.00399)	Respiratory therapists Per occupied bed 0.0025 (− 0.01044 to 0.01544) Physical therapist Per occupied bed − 0.0033 (− 0.02467 to 0.01807) Physician assistant Per occupied bed: 0.0298 (− 0.00255 to 0.06215) Pharmacists Per occupied bed − 0.0381* (− 0.06045 to − 0.01575) Occupational therapists Per occupied bed 0.0193 (− 0.01697 to 0.05557) Dietitians Per occupied bed − 0.0164 (− 0.04542 to 0.01262) Medical technologists per occupied bed: − 0.0086* (− 0.01625 to − 0.00095) Administrative staff Per occupied bed 0.0069* (0.00082 to 0.01298) Social workers Per occupied bed 0.0005 (− 0.02146 to 0.02246)
Checkley et al., 2014	ICU mortality	Hospital	% difference in mortality (95%CI)	Bed-to-nurse per 1:1 unit increase 3.7* (0.5 to 6.8)		Bed-to-residents/fellows per 1:1 unit increase 2.7 (− 3.8 to 9.1)	
Chung et al., 2018	All-cause mortality for stroke patients	Hospital	OR (95%CI)	Per 100 beds 0.988* (0.977 to 0.999)		Per 100 beds 0.988 (0.971 to 1.005)	
Griffiths et al., 2016	All-cause mortality for medical admissions	Hospital	RR (95%CI)	Hospital level: Occupied beds per FTE RN medical 1.14 (0.95 to 1.38) / surgical 0.94 0.73 (1.20 to 0.59) Unit Level: ≤ 6 patients per RN (vs ≥ 10) medical 0.89 (0.83 to 0.95)* surgical 0.89 (0.73 1.08)	Hospital level: occupied beds per FTE HCSW: medical 0.93 (0.89 to 0.98)* / surgical 0.95 (0.88 1.03)	Hospital level: Medical occupied beds per FTE doctor 1.08 (1.02 to 1.15)* / surgical 1.13 (1.04 to 1.22)	
Kim et al., 2020	Disease-specific (post-PCI mortality)	Hospital	OR (95%CI)	Bed per 1st grade RN 1.13 (0.63 to 2.03) Bed per 2nd grade 1.00 (0.73 to 1.35) Reference: 3rd grade		Adjusted for in models (no data presented)	
Neuraz et al., 2015	ICU mortality	Unit	RR (95%CI)	Patient to staff 1:1 to 1.5:1 = 1.9 (0.7 to 4.6) Patient to staff 1.5:1 to 2:1 = 2.0 (0.8 to 5.0) Patient to staff 2:1 to 2.5:1 = 2.3 (0.9 to 5.8) Patient to staff > 2.5:1 = 3.5* (1.3 to 9.1) Reference: patient to staff < 1:1		Patient to staff 8:1 to 10:1 = 0.9 (0.7 to 1.3) Patient to staff 10:1 to 14:1 = 1.1 (0.8 to 1.5) Patient to staff > 14:1 = 2.0* (1.3 to 3.2) Reference: patient to staff < 8:1	
Peelen et al., 2007	ICU mortality	Hospital	OR (95%CI)	Per ICU bed = 0.956 (0.861 to 1.063)		Intensivists per ICU bed = 1.164* (1.010 to 1.341)	
Ricciardi et al., 2014	All-cause mortality	Hospital	OR (95%CI)	Per bed category 0.75 to 1.359 = 0.9 (0.9 to 1.0) Per bed category ≥ 1.359 = 0.9* (0.9 to 0.9) Reference: per bed category 0 to 0.75		Per bed category 0.007 to 0.0067 = 0.9* (0.9 to 0.9) Per bed category ≥ 0.0067* (0.9 to 0.9) Reference: per category 0 to 0.007	
Robertson et al., 1999	Disease-specific (COPD)	Hospital	Annual coefficient	1989 coeff − 0.022 (− 0.505) 1990 coeff − 0.012 (− 0.295) 1991 coeff 0.013 (0.369)	1989 coeff − 0.080 (− 1.243) 1990 coeff − 0.081 (− 1.338) 1991 coeff 0.013 (− 0.240)	Physicians: 1989 coeff − 0.240 (− 1.492) 1990 coeff − 0.136 (− 1.363) 1991 coeff − 0.147 (− 1.181) Medical residents & interns: 1989 coeff − 0.060 (− 0.534) 1990 coeff − 0.094 (− 0.888) 1991 coeff 0.051 (− 0.595)	Ancillary nursing personnel: 1989 coeff − 0.0220 (− 0.461) 1990 coeff 0.040 (0.962) 1991 coeff 0.017 (0.477) Respiratory therapy technicians: 1989 coeff − 1.498* (− 3.495) 1990 coeff − 0.318 (− 1.012) 1991 coeff − 1.076* (− 3.995) Radiographers and radiologic technologists: 1989 coeff 0.459 (1.637) 1990 coeff 0.440 (1.700) 1991 coeff 0.128 (0.601) Respiratory therapists 1989 coeff − 0.310 (− 1.034) 1990 coeff − 0.622* (− 2.188) 1991 coeff − 0.622* (− 2.447) Physical therapists 1989 coeff 0.210 (0.402) 1990 coeff 0.516 (1.138) 1991 coeff 0.298 (0.738) Pharmacists 1989 coeff 0.716 (1.268) 1990 coeff − 0.725 (− 1.433) 1991 coeff 0.018 (0.041) Occupational therapists 1989 coeff − 0.694 (− 0.863) 1990 coeff 0.205 (0.300) 1991 coeff 0.653 (0.992) Dietitians 1989 coeff − 0.057 (− 0.096) 1990 coeff 1.589* (2.821) 1991 coeff 0.168 (0.254) Other laboratory personnel: 1989 coeff − 0.267 (− 1.451) 1990 coeff − 0.372* (2.299) 1991 coeff 0.187 (1.287) Radiation therapists: 1989 coeff − 0.329 (− 0.490) 1990 coeff 1.056 (1.469) 1991 coeff 0.704 (1.440) Nuclear medicine technologists: 1989 coeff − 2.274 (− 2.068) 1990 coeff − 1.807 (− 1.759) 1991 coeff − 0.607 (0.905) Other radiologic personnel: 1989 coeff 0.471 (1.758) 1990 coeff 0.223 (0.990) 1991 coeff − 0.177 (− 0.972) Medical technologists: 1989 coeff − 0.148 (− 0.769) 1990 coeff 0.039 (0.229) 1991 coeff 0.145 (1.021) Administrative staff: 1989 coeff 0.275 (1.685) 1990 coeff 0.217 (1.392) 1991 coeff 0.024 (0.204)
Smith et al., 2007	All-cause mortality (?)	Patient	OR (95%CI)	High RN-occupied bed OR 0.68 (0.40 to 1.17)	High licensed vocational nurse-occupied bed 2.00 (0.99 to 4.05)		High respiratory therapy-occupied bed 1.64 (0.92 to 2.94)
West et al., 2014	All-cause mortality	Unit	OR (95%CI)	Direct care nurses per bed OR 0.92 (0.86 to 0.98)*	Support staff per bed 1.08 (0.78 to 1.49)	“Half day” per bed Intensivist 0.99 [0.83 to 1.19] Consultants 0.90 [0.8 to 0.99]*	

*Statistically significant (*p* < 0.05)

### Risk of bias assessment

All risk of bias assessments are reported in Table 3. Four studies were classified with strong internal validity [25, 26, 30], and eight with moderate internal validity [20-24, 27-29]. Studies classified as stronger from an internal validity perspective were longitudinal, meaning that bias due to simultaneity was less likely to occur. All studies were ranked as strong in terms of reliability and completeness of outcome measurement because patient mortality was derived from administrative systems which are less prone to bias than, for example, surveys where outcomes are reported by individual respondents. Studies scored strongly in the confounding and methods domain when, in addition to robust risk-adjustment of patient mortality, they were able to take into account clustering of responses in units and hospitals, or at least one of the two [20, 24, 25, 30]. Confidence intervals, where reported, were generally narrow in absolute terms although absolute effects tended to be small and so proportionate changes in effects could still be large. Ten studies had strong external validity because of the large number of hospitals included giving the studies high power and representativeness in a defined administrative area [20, 22-25, 27-31], while two had moderate external validity [21, 26].

**Table 3:** Risk of bias assessment

Study	Design	Setting	Eligible population	Selected participants	Outcome measures reliable	Outcome measures complete	Sufficient powered	Appropriate confounding	Appropriate methods	Precision of association	Internal validity	External validity
Bjerregard et al., 2020	−	+	+ +	+ +	+ +	+ +	+	+ +	+ +	+	+	+ +
Bond et al., 1999	−	+	+ +	+ +	+ +	+ +	+ +	+	+	+ +	+	+ +
Checkley et al., 2014	−	+	+ +	+	+ +	+ +	+ +	+	+	+	+	+ +
Chung et al., 2018	−	+	+ +	+	+ +	+ +	+ +	+ +	−	+ +	+	+ +
Griffiths et al., 2016	−	+ +	+ / + + *	+ / + + *	+ +	+ +	+ +	+ +	+ +	+ +	+ +	+ +
Kim and Kim, 2020	−	+	+	+	+ +	+ +	+ +	+ +	+ +	+ +	+ +	+
Neuraz et al., 2015	+ +	+	+	+ +	+ +	+ +	+	+	+	+	+	+
Peelen et al., 2007	−	+	+ +	+ +	+ +	+ +	+ +	+	+	+ +	+	+ +
Ricciardi et al., 2014	−	+	+ +	+ +	+ +	+ +	+ +	+ +	+	+ +	+	+ +
Robertson and Hassan, 1999	−	+	+ +	+	+ +	+ +	+ +	+	+	−	+	+ +
Smith et al., 2007	−	+	+ +	+ +	+ +	+ +	+ +	+	+	+ +	+	+ +
West et al., 2014	−	+ +	+ +	+ +	+ +	+ +	+ +	+ +	+ +	+ +	+ +	+ +

^*^High risk of bias (weak), + moderate, +  + low risk of bias (strong) *Different rating for different aspects of the study (national vs selective sample)

### Nurse staffing levels

There was a statistically significant association (*p* < 0.05) between higher levels of registered nurse staffing and lower mortality rates in seven studies out of 12 [20-24, 27, 30]. The effect sizes were typically small and were difficult to compare because of the varying staffing measures (see Table 2). For example, an increase of 1 registered nurse hour per patient day reduced odds of death by less than 1% based on the reported beta coefficient of − 0.008 [20]. An additional nurse per bed reduced the absolute death rate by 0.26 [22]. An additional RN per 100 beds reduced the odds of death by 1% [24]. Odds of death were reduced by 10% when there were ≥ 1.359 registered nurses per bed compared to between 0 and 0.75 registered nurses per bed [27]. In ICU settings, an additional registered nurse per bed reduced the odds of death by 8% [30]. An increase of one in the bed-to-nurse ratio was associated with a 3.7% higher mortality rate [23]). A larger effect was observed in the longitudinal study by Neuraz et al. [21] in an ICU setting, where having more than 2.5 patients per registered nurses was associated with an almost fourfold increase in the risk of mortality (risk ratio = 3.5) compared to having less than 1 patient per registered nurse.

Although most analyses assumed a linear effect, those that categorised staffing levels across more than two categories found that Higher registered nurse staffing categories were associated with lower mortality and vice versa [21, 26, 27] although non-linearity was not formally assessed. There was evidence that estimated nurse staffing effects were lower in multivariable models controlling for other staff groups than in models including nurse staffing only. For example in Griffiths et al.’s study of English NHS hospitals, a reduction in the mean registered nurse workload from 10 or more patients to 6 or fewer was associated with a 20% reduction in the risk of death in the single staff group model which reduced to 11% in the model including medical staffing levels [25]. Five studies did not find statistically significant associations between registered nurse staffing levels and mortality, although in all cases point estimates were in the direction of a beneficial effect from higher levels of registered nurse staffing [25, 26, 28, 29, 31]. No studies found that hospitals with more registered nurses had higher mortality rates.

Six studies included nursing assistant staffing levels, with one finding a beneficial effect from higher staffing levels (odds ratio from β coefficient for hours per patient day (HPPD) = 0.99) [20], and two finding that higher nursing assistant staffing levels were associated with higher patient mortality risk (with a 0.4% absolute risk increase for each assistant per occupied bed [22] and occupied beds per nursing assistant OR = 0.93 [25]). The three remaining studies did not report statistically significant associations, but estimates, where available, pointed to higher staffing levels being associated with higher mortality [29, 30].

### Physician staffing levels

Eleven studies reported associations with physician staffing levels. Of these, seven found that higher levels of physician staffing were statistically significantly associated with lower hospital mortality rates, after adjusting for nurse staffing levels [20-22, 25, 27, 30, 31]. Effect sizes tended to be small, apart from Neuraz et al., where the risk of mortality doubled when having more than 14 patients per physician compared to having less than 8 patients per physician [21]. When adding one physician per bed, effect sizes were odds ratio = 0.99 [20, 22] and having more than 1.359 physicians per bed compared to between 0 and 0.75 physicians per bed was associated with a 10% reduction in the likelihood of a patient dying [27]. When adding one bed per physician, the likelihood of patients dying increased by 8% [25] and 16% [31]. Estimates from other studies were also small and not statistically significant but all were in the direction of a protective effect from having more physicians per bed [23, 24, 30]. In one instance, claims of no associations meant that analyses were not reported [26]. One study compared different physician grades (i.e. intensivists vs consultants), but none of these staff groups were associated with mortality [30]. One study included physician assistants, and, while estimates indicated that higher staffing levels were associated with lower mortality, these were not statistically significant [22].

### Other staff groups

Only two studies reported on staff groups other than medical and nursing staff (Table 1). Robertson and colleagues, analysing data from 1791 US hospitals over 3 years (1989–1991), considered (in addition to nurses, nursing assistants, and physicians) respiratory therapists; physical therapists; pharmacists; occupational therapists; laboratory staff; dietitians; medical technologists; administrative staff; and social workers. They found that higher levels of staff employed per 100 adjusted admissions were significantly associated with lower mortality rates from chronic obstructive pulmonary disease (COPD) for respiratory therapists (odds ratio from *β* coefficient = 0.53), respiratory therapy technicians (odds ratio from *β* coefficient = 0.22), and laboratory staff (odds ratio from *β* coefficient = 0.68). Associations for other staff groups were not statistically significant [28].

Bond et al. analysed 1992 data from 3763 US hospitals and included (in addition to nurses, nursing assistants and physicians, and physician assistants) respiratory therapists; physical therapists; respiratory therapy technicians; radiographers and radiologic technologists; pharmacists; occupational therapists; dietitians; radiation therapists; nuclear medicine technologists; medical technologists; administrative staff; and social workers. Of these, they found statistically significant associations between more pharmacists per bed (OR from *β* coefficient = 0.97) and medical technologist staff per bed (*β* coefficient = 0.99) and lower mortality rates, while hospitals with more administrative staff per bed had higher hospital mortality (*β* coefficient = 0.006). Associations for other staff groups were not statistically significant [22].

## Discussion

This is the first literature review to synthesise evidence of associations between patient mortality and multi-disciplinary hospital staffing. Having more physicians and registered nurses was associated with lower mortality, and higher levels of nursing assistants were associated with higher patient mortality. Only two studies reported associations with other staffing groups, finding statistically significant associations between higher pharmacists and medical technologists staffing and lower mortality in one study and higher laboratory staff, respiratory therapists and respiratory therapy technicians and lower mortality from COPD in another. While data in these studies are drawn from thousands of hospitals, the data are now over 30 years old, and the roles and responsibilities of staff groups are likely to have changed substantially since then, so the extent to which these findings generalise to current contexts is questionable.

For all staff groups, beneficial effects for patients potentially extend far beyond reducing the risk of death. Occupational groups such as physiotherapists, nutritionists, and occupational therapists play an important role in hospitals in providing early mobilisation and/or adequate nutrition, and improving functional ability and activities of daily living [32, 33] although the limited evidence hampers any conclusion.

The finding that physician staffing levels were associated with patient risk of death is not surprising, as physicians, are, in general, the main decision-makers when it comes to patients’ care pathways and treatments, and the relationship we found is plausibly causal. Nurse staffing levels and physician staffing levels tend to be strongly correlated [34] and so it is possible that associations between nurse staffing and mortality in studies that omit physician staffing are partly attributable to medical staffing levels. Nonetheless, nurse staffing levels were associated with mortality after controlling for physicians in most studies and so the possibility that there is no independent nurse staffing effect can be discounted. The finding that having higher levels of nursing assistants was associated with higher mortality in most studies mirrors that of studies focusing on nursing only [35]. The reasons for an adverse effect from additional nursing support staff are complex, but suggested mechanisms include excessive substitution of assistants for registered nurses and insufficient registered nurses to properly supervise assistants [36].

Most studies used data from large patient samples from multiple hospitals across several years, but analyses were often cross-sectional, and associations measured at the hospital level, whereby staffing over one year was averaged and related to the average mortality rate for that same year. This level of aggregation and analysis means that estimates could still be biased by endogeneity, in particular the simultaneity bias [37] whereby hospitals with more acutely ill patients, who also have higher mortality risk, may have higher staffing levels to meet patient demand. Although risk adjustment makes this an unlikely explanation of results, estimates of effect could still be attenuated. Aggregating staffing levels in the form of bed-to-staff employed or employed staff-to-bed at the hospital level also masks considerable variation between units and from day to day, which again would tend to attenuate estimated adverse effects from staffing variation.

In recent years, the evidence base around nurse staffing levels has advanced substantially thanks to longitudinal studies analysing routinely collected data, which allow exploration of associations at the ward level or even at the patient level [6]. Nonetheless, the availability and quality of such data for other staff groups is currently unknown. Future studies using data extracted from nursing rosters should simultaneously explore the availability of roster data of other staff groups. Such studies have the potential to enhance the quality of the evidence base to guide policy-makers and those in charge of planning the health workforce nationally and locally.

### Limitations

We produced an extensive search strategy, but it is possible that we did not capture all studies due to the complexity of the topic and the vast number of existing healthcare professional figures. Nonetheless, it is unlikely that we would have missed a sufficient number of recent studies to change our conclusions.

## Conclusions

The association between higher nurse staffing levels and reduced mortality stands also when controlling for other staff groups, highlighting that the research and policy endeavour around nurse staffing is justified and necessary. Nonetheless, physicians’ staffing levels are also associated with patients’ risk of death, although the evidence is sparse and, while professional bodies globally produced standards and guidelines, no policy directly addresses how to appropriately staff services with physicians. The picture for other staff groups becomes even blurrier, as the evidence for other staffing groups is both scant and unclear, although the pathways for such staffing groups to impact patient outcomes are plausible and should be further explored in future studies, possibly including other outcomes in addition to mortality. The role of occupational groups such as physiotherapists, occupational therapists, dietitians, pharmacists, and other clinical staff should not be discounted based on absence of evidence of an effect on patient mortality. Future research and policy should strive to address this gap to ensure safe staffing is achieved for all professional groups in hospital.

## Supplementary Information

**Additional file 1: File S1.** Search terms. **File S2.** Risk of bias assessment checklist, adapted from Griffiths et al. [18].

## Data Availability

Not applicable.

## References

[1] Buchan J, Gershlick B, Charlesworth A, Seccombe I. Falling short: the NHS workforce challenge. The Health Foundation. 2019.

[2] Rocks S, Boccarini G, Charlesworth A, Idriss O, McConkey R, Rachet-Jacquet L. Health and social care funding projections. 2021. The Health Foundation; 2021.

[3] World Health Organization. Global strategy on human resources for health: workforce 2030. 2016.

[4] World Health Organization (2022). The 2022 update.

[5] Kane RL, Shamliyan TA, Mueller C, Duval S, Wilt TJ (2007). The association of registered nurse staffing levels and patient outcomes: systematic review and meta-analysis. Med Care.

[6] Dall'Ora C, Saville C, Rubbo B, Turner L, Jones J, Griffiths P (2007). Nurse staffing levels and patient outcomes: a systematic review of longitudinal studies. Int J Nurs Stud..

[7] Numata Y, Schulzer M, Van Der Wal R, Globerman J, Semeniuk P, Balka E (2006). Nurse staffing levels and hospital mortality in critical care settings: literature review and meta-analysis. J Adv Nurs.

[8] Rae PJL, Pearce S, Greaves PJ, Dall'Ora C, Griffiths P, Endacott R (2021). Outcomes sensitive to critical care nurse staffing levels: a systematic review. Intensive Crit Care Nurs.

[9] McHugh MD, Kelly LA, Sloane DM, Aiken LH (2011). Contradicting fears, California's nurse-to-patient mandate did not reduce the skill level of the nursing workforce in hospitals. Health Aff (Millwood).

[10] Gerdtz MF, Nelson S (2007). 5-20: a model of minimum nurse-to-patient ratios in Victoria, Australia. J Nurs Manag.

[11] Van den Heede K, Cornelis J, Bouckaert N, Bruyneel L, Van de Voorde C, Sermeus W (2020). Safe nurse staffing policies for hospitals in England, Ireland, California, Victoria and Queensland: a discussion paper. Health Policy.

[12] McHugh MD, Aiken LH, Sloane DM, Windsor C, Douglas C, Yates P (2021). Effects of nurse-to-patient ratio legislation on nurse staffing and patient mortality, readmissions, and length of stay: a prospective study in a panel of hospitals. Lancet.

[13] Butler R, Monsalve M, Thomas GW, Herman T, Segre AM, Polgreen PM (2018). Estimating time physicians and other health care workers spend with patients in an intensive care unit using a sensor network. Am J Med.

[14] Griffiths P, Ball J, Drennan J, Dall’Ora C, Jones J, Maruotti A (2016). Nurse staffing and patient outcomes: strengths and limitations of the evidence to inform policy and practice. A review and discussion paper based on evidence reviewed for the National Institute for Health and Care Excellence Safe Staffing guideline development. Int J Nurs Stud.

[15] Schoo AM, Boyce RA, Ridoutt L, Santos T (2008). Workload capacity measures for estimating allied health staffing requirements. Aust Health Rev.

[16] Lee H, Ryu K, Sohn Y, Kim J, Suh GY, Kim E (2019). Impact on patient outcomes of pharmacist participation in multidisciplinary critical care teams: a systematic review and meta-analysis. Crit Care Med.

[17] Sabin J, Subbe CP, Vaughan L, Dowdle R (2014). Safety in numbers: lack of evidence to indicate the number of physicians needed to provide safe acute medical care. Clin Med.

[18] Griffiths P, Ball J, Drennan J, James L, Jones J, Recio A, et al. The association between patient safety outcomes and nurse/healthcare assistant skill mix and staffing levels and factors that may influence staffing requirements. Project Report. 2014.

[19] National Institute for Health and Care Excellence (2012). Methods for the development of NICE public health guidance.

[20] Bjerregaard U, Hølge-Hazelton B, Rud Kristensen S, Rose Olsen K. Nurse staffing and patient outcomes: analyzing within-and between-variation. DaCHE discussion papers. 2020;3.

[21] Neuraz A, Guérin C, Payet C, Polazzi S, Aubrun F, Dailler F (2015). Patient mortality is associated with staff resources and workload in the ICU: a multicenter observational study. Crit Care Med.

[22] Bond C, Raehl CL, Pitterle ME, Franke T (1999). Health care professional staffing, hospital characteristics, and hospital mortality rates. Pharmacotherapy J Hum Pharmacol Drug Therapy..

[23] Checkley W, Martin GS, Brown SM, Chang SY, Dabbagh O, Fremont RD (2014). Structure, process and annual intensive care unit mortality across 69 centers: United States Critical Illness and Injury Trials Group Critical Illness Outcomes Study (USCIITG-CIOS). Crit Care Med.

[24] Chung W, Sohn M (2018). The impact of nurse staffing on in-hospital mortality of stroke patients in Korea. J Cardiovasc Nurs.

[25] Griffiths P, Ball J, Murrells T, Jones S, Rafferty AM (2016). Registered nurse, healthcare support worker, medical staffing levels and mortality in English hospital trusts: a cross-sectional study. BMJ Open.

[26] Kim Y, Kim J (2020). In-hospital mortality in patients receiving percutaneous coronary intervention according to nurse staffing level: an analysis of National Administrative Health Data. Int J Environ Res Public Health.

[27] Ricciardi R, Nelson J, Roberts PL, Marcello PW, Read TE, Schoetz DJ (2014). Is the presence of medical trainees associated with increased mortality with weekend admission?. BMC Med Educ.

[28] Robertson RH, Hassan M (1999). Staffing intensity, skill mix and mortality outcomes: the case of chronic obstructive lung disease. Health Serv Manage Res.

[29] Smith DL, Elting LS, Learn PA, Raut CP, Mansfield PF (2007). Factors influencing the volume–outcome relationship in gastrectomies: a population-based study. Ann Surg Oncol.

[30] West E, Barron DN, Harrison D, Rafferty AM, Rowan K, Sanderson C (2014). Nurse staffing, medical staffing and mortality in intensive care: an observational study. Int J Nurs Stud.

[31] Peelen L, de Keizer NF, Peek N, Scheffer GJ, van der Voort PH, de Jonge E (2007). The influence of volume and intensive care unit organization on hospital mortality in patients admitted with severe sepsis: a retrospective multicentre cohort study. Crit Care.

[32] Weinreich M, Herman J, Dickason S, Mayo H (2017). Occupational therapy in the intensive care unit: a systematic review. Occup Therapy Health Care.

[33] Hanekom SD, Faure M, Coetzee A (2007). Outcomes research in the ICU: an aid in defining the role of physiotherapy. Physiother Theory Pract.

[34] Griffiths P, Jones S, Bottle A (2013). Is, “failure to rescue” derived from administrative data in England a nurse sensitive patient safety indicator for surgical care? Observational study. Int J Nurs Stud.

[35] Griffiths P, Maruotti A, Recio Saucedo A, Redfern OC, Ball JE, Briggs J (2019). Nurse staffing, nursing assistants and hospital mortality: retrospective longitudinal cohort study. BMJ Qual Saf.

[36] Griffiths P, Ball J, Bloor K, Böhning D, Briggs J, Dall’Ora C (2018). Nurse staffing levels, missed vital signs and mortality in hospitals: retrospective longitudinal observational study. Health Serv Deliv Res..

[37] Antonakis J, Bendahan S, Jacquart P, Lalive R (2010). On making causal claims: a review and recommendations. Leadersh Quart.

