# Supplementary material for: The association between multi-disciplinary staffing levels and mortality in acute hospitals: a systematic review
Source: Hum Resour Health. 2023 Apr 20;21:30. doi: 10.1186/s12960-023-00817-5 (PMC10116759; doi:10.1186/s12960-023-00817-5)
Supplement: Supplementary file 1 — Additional file 1: File S1. Search terms. File S2. Risk of bias assessment checklist, adapted from Griffiths et al. [18]. [file 12960_2023_817_MOESM1_ESM.docx]

# File S1 Full search terms

**FACET A: Staffing groups and staffing levels**

1. (workload* or workforce* or shift or shiftwork* or shifts or overtime).ab,ti. (819768)

2. (Skill mix$ or skillmix or skill-mix$ or skills mix).ab,ti. (2381)

3. (skill* adj1 mix).ab,ti. (2311)

4. "Staff* level$".ab,ti. (5176)

5. exp Work schedule/ (50578)

6. exp "Personnel Staffing and Scheduling"/ (135084)

7. exp Health manpower/ (14517)

8. exp Workload/ (67946)

9. "skillmix*".ab,ti. (22)

10. (staffmix* or "staff mix*").ab,ti. (215)

11. staffing.ab,ti. (33878)

12. "understaff*".ab,ti. (1445)

13. "under staff* ".ab,ti. (175)

14. (staff* adj3 (level* or ratio* or resourc* or model* or number* or mix* or rota* or rosta* or roster* or schedul* or overtime or supervision or supervisory)).ab,ti. (288992)

15. (staff* adj3 (sufficient* or sufficiency or adequate* or adequac* or target* or insufficient* or insufficienc* or inadequate* or inadequac* or short or shortage or efficient* or efficienc* or inefficien*)).ab,ti. (10874)

16. (Doctor? adj5 (number or workforce or workforce or staffing or workload or skill mix)).ab,ti. (4617)

17. (Medical staff* adj5 (number or workforce or workforce or staffing or workload or skill mix)).ab,ti. (924)

18. (Physician? adj5 (number or workforce or work force)).ab,ti. (14754)

19. (surgeon? adj5 (number or workforce or workforce or staffing or workload or skill mix)).ab,ti. (5883)

20. hospital? volunteer$.ab,ti. (324)

21. hospital? assistant?.ab,ti. (57)

22. (consultant? adj5 (number or workforce or workforce or staffing or workload or skill mix)).ab,ti. (987)

23. (Pharmacist? adj5 (number or workforce or workforce or staffing or workload or skill mix)).ab,ti. (2450)

24. (Physiotherap* adj5 (number or workforce or workforce or staffing or workload or skill mix)).ab,ti. (842)

25. (Occupational therap* adj5 (number or workforce or workforce or staffing or workload or skill mix)).ab,ti. (319)

26. (Health* professional? adj5 (number or workforce or workforce or staffing or workload or skill mix)).ab,ti. (1613)

27. (Allied health professional? adj5 (number or workforce or workforce or staffing or workload or skill mix)).ab,ti. (74)

28. "Health* professional?".ab,ti. (183749)

29. 1 or 2 or 3 or 4 or 5 or 6 or 7 or 8 or 9 or 10 or 11 or 12 or 13 or 14 or 15 or 16 or 17 or 18 or 19 or 20 or 21 or 22 or 23 or 24 or 25 or 26 or 27 or 28 (1211903)

**FACET B: Hospital setting**

30. exp Hospitals/ (1530104)

31. exp Hospital Units/ (704488)

32. exp Inpatients/ (209557)

33. Inpatient?.ab,ti. (296807)

34. Hospital?.ab,ti. (2982303)

35. (medical adj3 (unit* or ward*)).ab,ti. (42125)

36. (surgical adj3 (unit* or ward*)).ab,ti. (32162)

37. (patient* adj3 surgical).ab,ti. (243973)

38. (patient* adj3 medical).ab,ti. (184973)

39. ("medical-surgical" or "surgical-medical").ab,ti. (18135)

40. 30 or 31 or 32 or 33 or 34 or 35 or 36 or 37 or 38 or 39 (4230894)

**FACET C: Mortality**

41. Hospital Mortality/ (73896)

42. "hospital death?".ab,ti. (22951)

43. "mortality rate?".ab,ti. (345804)

44. "death rate?".ab,ti. (54129)

45. 41 or 42 or 43 or 44 (465117)

**COMBINE FACETS A-C**

46. 29 and 40 and 45 (4618)

47. limit 46 to humans **(4222)**

**Duplicates results**

541 duplicates removed

**Final results: 3681 abstracts**

# File S2 Risk of Bias assessment checklist, adapted from Griffiths et al, 2014

| **Reviewer** |  | | |
| --- | --- | --- | --- |
| **Study full ref** |  | | |
| **Short ID** |  | | |
|  | **Scores** | **Internal** | **External** |
|  | 2 strong (++) |  |  |
|  | 1 moderate (+) |  |  |
|  | 0 weak (-) |  |  |
| **Design** |  |  |  |
| Study design & analysis cross sectional (0) or allows for cause / effect (exposure precedes outcome) 2 |  |  |  |
|  |  |  |  |
| **Is the setting applicable to the UK?** |  |  |  |
| Did the setting differ significantly from the UK? |  |  |  |
| - UK ++ |  |  |  |
| - Other developed countries + |  |  |  |
| - Other - |  |  |  |
|  |  |  |  |
| **Is the eligible population or area representative of the source population or area?** |  |  |  |
| - Consider whether hospitals potentially included in the study are representative of acute general hospitals in that country / state (+1) |  |  |  |
| - Were the wards/ staff / patients eligible to be included in the hospitals representative of general / medical and / or surgical units [census/ stratified / random samples of med / surg units or patients] (+1) |  |  |  |
|  |  |  |  |
| **Do the selected participants or areas represent the eligible population or area?** |  |  |  |
| - What % of selected hospitals agreed to participate (+1) |  |  |  |
| - What % of eligible individuals (staff / patients) participated (60% + is acceptable)?(+1) |  |  |  |
| - Was the data derived from administrative systems and complete (+1) |  |  |  |
| - Were the inclusion or exclusion criteria explicit and appropriate? |  |  |  |
|  |  |  |  |
| **Were the outcome measures and procedures reliable?** |  |  |  |
| - Were main patient outcome measures subjective or objective (++ for objective measures) |  |  |  |
| - How reliable were outcome measures (e.g. inter- or intra-rater reliability scores)? |  |  |  |
| - Was there any indication that measures had been validated (e.g. validated against a gold standard measure or assessed for content validity)? |  |  |  |
|  |  |  |  |
| **Were the outcome measurements complete?** |  |  |  |
| - Were all or most of the study participants who met the defined study outcome definitions likely to have been identified? (++ for mortality, + for other PSIs collected using clearly defined methods, - if abstracted from discharge abstracts) |  |  |  |
|  |  |  |  |
| **Was the study sufficiently powered to detect an effect (if one exists)?** |  |  |  |
| - Were there sufficient units / hospitals / wards to give variation and enough patients to detect effects |  |  |  |
| - Large multi-hospital (20+) studies (state / national / international) with administrative data ++ |  |  |  |
| - Smaller studies / single hospital with large numbers of patients (000,000) + |  |  |  |
| - Other - |  |  |  |
|  |  |  |  |
| **How well were likely confounding factors identified and controlled for?** |  |  |  |
| - For main patient outcomes. Was there patient level risk adjustment for patient AGE, DIAGNOSIS and COMORBIDITY(++) |  |  |  |
| - For falls rates etc was there stratification by unit type (+) |  |  |  |
|  |  |  |  |
| **Were the analytical methods appropriate?** |  |  |  |
| - Was there adjustment for clustering of data within wards / hospitals? (+ 1) |  |  |  |
| - Where relevant was there control for ward / hospital characteristics? (+1) |  |  |  |
|  |  |  |  |
| **Was the precision of association given or calculable? Is association meaningful?** |  |  |  |
| - Were confidence intervals or p values for effect estimates given or possible to calculate? |  |  |  |
| - Were CIs wide or were they sufficiently precise to aid decision-making? If precision is lacking, is this because the study is under-powered? |  |  |  |
|  |  | **Overall internal validity** | **Overall external validity** |
|  |  |  |  |
| **Are the study results internally valid (i.e. unbiased)?** |  |  |  |
| - How well did the study minimise sources of bias (i.e. adjusting for potential confounders)? |  |  |  |
| - Were there significant flaws in the study design? |  |  |  |
|  |  |  |  |
| **Are the findings generalisable to the source population (i.e. externally valid)?** |  |  |  |
| - Are there sufficient details given about the study to determine if the findings are generalisable to the source population? |  |  |  |
| - Consider: participants, interventions and comparisons, outcomes, resource and policy implications. |  |  |  |
